# Information and Communications Technology-Based Telehealth Approach for Occupational Therapy Interventions for Cancer Survivors: A Systematic Review

**DOI:** 10.3390/healthcare8040355

**Published:** 2020-09-23

**Authors:** Na-Kyoung Hwang, Young-Jin Jung, Ji-Su Park

**Affiliations:** 1 Department of Occupational Therapy, Seoul North Municipal Hospital, Seoul 02062, Korea; occupation81@naver.com; 2 Department of Radiological Science at Health Sciences Division DongSeo University, Busan 47011, Korea; 3 Advanced Human Resource Development Project Group for Health Care in Aging Friendly Industry, Dongseo University, Busan 47011, Korea

**Keywords:** cancer survivor, occupational therapy, systematic review, telehealth

## Abstract

(1) Background: Occupational therapy (OT) practice has a unique perspective that addresses the complex needs of cancer survivors. Despite the expanded research and application of OT services using telehealth (TH) to promote clients’ health and well-being, studies on OT services using TH for cancer survivors are rare. This study aimed to review the TH approaches in the scope of OT and the outcome of factors affecting occupational engagement in adult cancer survivors. (2) Materials and Methods: This systematic review performed a literature search of five databases (Medline Complete, PubMed, CINAHL, PsycINFO, Web of Science) using a combination of keywords and cross-referencing. Studies were included if they described a TH intervention within the scope of OT practice to improve occupational engagement. (3) Results: Fifteen studies (12 randomized controlled trials, three quasiexperimental studies) were reviewed. Physical activity had a positive effect on physical and cognitive function. Symptom self-management showed positive effects on the relief of symptom burden. Psychosocial interventions, which included cognitive behavioral therapy, problem-solving, cognitive behavioral therapy for insomnia, mind–body training, reduced sleep disturbance, and improved physical activity. Lifestyle behavior change interventions improved participation in moderate-intensity physical activity and diet quality. In addition, these interventions reduced cancer-related symptoms such as pain, depression, fatigue, distress, and improved quality of life. There were no direct outcomes of occupational engagement, excluding sleep, that could be confirmed through this review. (4) Conclusion: This review explored and confirmed the usefulness of TH approaches in the scope of OT practice in adult cancer survivors. It also supports the notion that OT-specific research using TH interventions for cancer survivors will be needed in the future.

## 1. Introduction

According to the Centers for Disease Control and Prevention [1], a cancer survivor is defined as “a person who has been diagnosed with cancer from the time of diagnosis throughout his or her life”. The transition from the primary treatment of cancer to aftercare is a difficult process that changes the survivors’ daily lives and requires continuous medical care [2]. As a result of the treatment progress, the survival rate increases, but the survivors have physical and psychosocial sequelae, which limit meaningful activity and participation during survivorship [3]. Common sequelae experienced by cancer survivors include depression, anxiety, fear of recurrence, cognitive dysfunction, fatigue, pain, peripheral neuropathy, reduced mobility and weakness, lymphedema, dysphagia, stoma care, bladder, and bowel dysfunction. These conditions prevent survivors from participating in meaningful activities and life occupations during survivorship, therefore, deteriorating the survivors’ quality of life (QOL) [4,5]. Cancer survivorship is classified as a chronic condition given that the complications and sequelae often last long [6].

Occupational therapy (OT) practitioners have a unique perspective on the client’s complex functional needs and provide services focused on improving the client’s health, well-being, and functional skills [7]. OT for cancer survivors can facilitate the improvement of functional levels and meaningful occupational performance (basic activities of daily living (BADL), instrumental activity of daily living (IADL), work, leisure, and social participation) during survivorship [8].

“Telehealth (TH)” is a potential occupational therapy service delivery model for cancer survivors. It is a new approach by means of information technology (IT), which connects health care centers and clients in remote areas via telecommunication. It is used mainly for consultations, training, supervision, and educational purposes [9,10]. In a survey on the use of telehealth usage for occupational therapists in Houston, USA, 22% of all respondents reported the use of telehealth for evaluation and intervention [11]. Assessments were used for ADL as well as neuromuscular and assistive technology, and interventions mainly included ADL training, environmental modification, and patient consultation. Although issues related to reimbursement still remain, many studies support the use of telehealth as one of the service delivery models in occupational therapy [12,13]. American Occupational Therapy Association (AOTA) is recommended TH to health organizations for OT for the following reasons: (1) TH can be applied to general health, psychological well-being, mental health, and motor rehabilitation in the OT field [14]; (2) OT services using TH can be provided in a “synchronous” way through real-time interaction or in an “asynchronous” way using store-and-forward technology; and (3) it can be performed where the client lives, works, learns, and plays if needed or desired [15]. 

Recently, the use of TH in OT service provision to promote the client’s evaluation and self-management, home environment modification, cognitive function, physical function, and disability prevention have been reported [16]. Hegel et al. reported that telephone-delivered problem-solving OT intervention can help reduce participation restrictions of breast cancer survivors in rural areas [17]. However, despite the unique perspective of OT practice dealing with the complex needs of cancer survivors and the expansion of research and application of OT services using TH, research on OT practice using TH for cancer survivors are rare. Therefore, the purpose of this study was to search and review the interventions within the scope of OT practice to improve occupational engagement. Our main study question was, “What are the evidences for TH intervention within the scope of OT that addressed the cancer survivors’ needs of activity and participation in BADL, IADL, work, leisure, social participation, and rest and sleep?”

## 2. Materials and Methods 

This systematic review was guided by the Preferred Reporting Items for Systematic Reviews and Meta-Analyses (PRISMA) statement [18].

### 2.1. Data Acquisition

An electronic database search was conducted from inception to 20 July 2020 using the following databases: Medline Complete (Ebsco Health), PubMed, CINAHL, PsycINFO, Web of Science. The following keywords were used: “telehealth”, “telerehabilitation”, “telemedicine”, “e-health”, “mobile health”, AND “cancer survivor”. For factors affecting occupational engagement, we used “quality of life”, “pain”, “fatigue”, “depression”, “physical activity”, “cognition”, “symptom burden”, “quality of life (QOL)”, OR “activities of daily living”, “work”, “leisure”, “social participation”, “sleep”. After the initial search, study titles and abstracts were examined. We went further to obtain the full text of eligible studies based on the inclusion and exclusion criteria. Manuscripts were then further examined for inclusion and exclusion criteria postretrieval. A consensus among all the authors was sought for a study’s final inclusion in the systematic review.

### 2.2. Eligibility Criteria 

We included English peer-reviewed journals within the scope of OT practice published between 2011 and 2020 which met the following inclusion criteria: (1) adult cancer survivors aged 18 years or older; (2) survivors had completed primary cancer treatment (surgery plus adjuvant chemotherapy and/or radiation therapy); (3) studies using TH interventions including telephone calls and/or web- or mobile-app-based interventions; (4) studies with outcomes focused on physical, cognitive, and psychosocial factors affecting occupational engagement, symptom burden, QOL, and emotional factors affecting confidence, resilience, and self-control; and (5) studies with outcome focused on activity and participation in BADL, IADL, work, leisure, social participation, and rest and sleep.

The following studies were excluded: (1) studies involving childhood cancer survivors, (2) studies focusing on caregivers or family, (3) studies on the effectiveness of palliative care, (4) studies involving patients on active cancer treatment, and (5) dissertations, theses, and protocol studies.

## 3. Results

### 3.1. Study Selection

**Figure 1:**
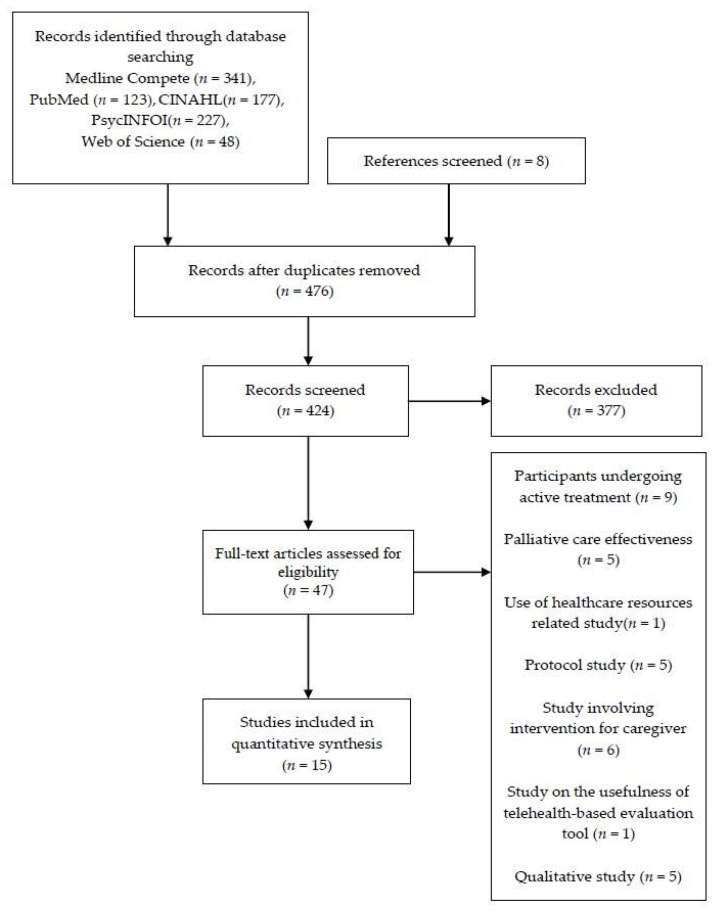
Study selection flow diagram and number of studies reviewed.

Figure 1 is a flow diagram of the study selection process. 

Nine hundred and twenty-four studies were identified from five databases: Medline Complete (*n* = 341), PubMed (*n* = 123), CINAHL (*n* = 177), PsycINFOI (*n* = 227), Web of Science (*n* = 48), and cross-referencing (*n* = 8). Only 15 studies fulfilled all the selection criteria. Two reviewers screened the full texts independently. 

### 3.2. Quality Assessment

The randomized controlled trials (RCTs) were rated using the Physiotherapy Evidence Database (PEDro) Scale [19]. Ten out of 12 RCTs were rated as “High” quality but showed low scores in the following scale elements: concealed allocation, blind subjects, blind therapists, and blind assessors. Two studies were rated as “fair” quality but showed low scores in concealed allocation, blind subjects, blind therapists, blind assessors, adequate follow-up, and intention-to-treat analysis (Table 1).

### 3.3. Participants’ Characteristics

The 15 studies comprised 2688 participants in total. Sample sizes varied widely across the studies, ranging from 18 [20] to 556 participants [21]. The studies focused on the following cancer types: breast cancer (*n* = 9), breast cancer and endometrial cancer (*n* = 1), prostate cancer (*n* = 1), hematological cancer (*n* = 1), and mixed cancer (*n* = 3; Table 2).

**Table 1:** PEDro scale scores for each study.

Author (Year)	Eligibility	Random Allocation	Concealed Allocation	Baseline Comparability	Blind Subjects	Blind Therapists	Blind Assessors	Adequate Follow-Up	Intention-to-Treat Analysis	Between-Group Comparisons	Point Estimated Variability	Score	Quality
Skolarus et al. (2019) [21]	Yes	1	0	1	0	0	0	1	1	1	1	6/10	High
Kim et al. (2011) [22]	Yes	1	0	1	0	0	0	1	1	1	1	6/10	High
Galiano-Castillo et al. (2016) [23]	Yes	1	1	1	0	0	1	1	1	1	1	8/10	High
Meneses et al. (2018) [24]	Yes	1	0	1	0	0	0	1	1	1	1	6/10	High
Frensham et al. (2018) [25]	Yes	0	0	1	0	0	1	1	1	1	1	6/10	High
Willems et al. (2017) [26]	Yes	1	0	1	0	0	0	1	1	1	1	6/10	High
Freeman et al. (2015) [27]	Yes	1	0	1	0	0	0	1	1	1	1	6/10	High
Zachariae et al. (2018) [28]	Yes	1	1	1	0	0	0	1	1	1	1	7/10	High
Kanera et al. (2017) [29]	Yes	1	0	1	0	0	0	0	1	1	1	5/10	Fair
Galiano-Castillo et al. (2017) [30]	Yes	1	1	1	0	0	1	1	1	1	1	8/10	High
Syrjala et al. (2018) [31]	Yes	1	0	1	0	0	0	0	0	1	1	4/10	Fair
Lee et al. (2014) [32]	Yes	1	1	1	0	0	0	1	1	1	1	7/10	High

PEDro Scale: Physiotherapy Evidence Database Scale.

**Table 2:** Summary of studies investigating the use of TH in the scope of OT practice.

Author (Year)	Study Design	Type of Cancer Size (Intervention/Control)	Intervention Group				Control Group
			Content (Care Model) TH Type	Intervention Activities	Delivery	Regime	
McCarthy et al. (2018) [20]	Quasi-experimental one group prepost	Breast Cancer 18	CBTI (Tele-education) Synchronous	• Online CBTI program • Reviewing sleep diaries and adjusting sleep schedules reinforced by education	Web	• 6 sessions for 6 weeks • 30–60 min for each session	No
Skolarus et al. (2019) [21]	RCT	Prostate cancer 278/278	Symptom self-management (Tele-education) Synchronous	• Self-management guidance through a series of tailored newsletters • Chosen symptom and self-management strategy suggestions	Tel	• 4 months • 1 time/month • 30 min call	Usual care • Nontailored newsletter about symptom management
Kim et al. (2011) [22]	RCT	Breast Cancer 23/22	Lifestyle change (Tele-counseling) Synchronous	• Counseling stage-matched exercise and diet intervention + workbook	Tel	• 3 months • a weekly basis • 30 min for telephone session	• Usual care
Galiano-Castillo et al. (2016) [23]	RCT	Breast Cancer 40/41	Exercise program (Tele-education) Synchronous and asynchronous	• Internet-based tailored exercise program and monitoring/comments the exercise feedback through videoconference	Web	• 8 weeks Exercise • 3 sessions/weeks (nonconsecutive) Videoconference • 3 times/week • 90 min/d	Usual care • Basic exercise recommendations (written format)
Meneses et al. (2018) [24]	RCT	Breast Cancer 21/19	Symptom self-management (Tele-education) Synchronous	Support and early education (Education sessions in the 1st month) • Education Common concerns among BCS and emphasized self-management techniques • Education binder and tip sheets • Support call Reinforcing self-management of health and understanding of side effects	Tel	• 3 months Education session • 45 min Support session • 60 min	Support and delayed education (education sessions in the 6th month)
Frensham et al. (2018) [25]	RCT	Mixed cancer types 51/51	Walking intervention (Tele-monitoring) Synchronous	• Provided lifestyle information and access to online resource • Performance self-monitoring using a pedometer • Setting individualized weekly step goals using RPE and achieving the goal + online forum to share experiences and offer peer support	Web	• 3 months •Daily monitoring	Wait list • Only provided with lifestyle information and a pedometer
Willems et al. (2017) [26]	RCT	Mixed cancer types 231/231	CBT + PST (Tele-education) Asynchronous	• Personalized advice and tailored information in psychosocial support and promoting positive lifestyle changes • Information of the common residual problems and self-management training	Web	• 6 months • 8 modules	Wait list • Care as usual
Freeman et al. (2015) [27]	3-armed RCT	Breast Cancer LD 48/TD 23/Control 47	MBT (Tele-education) Synchronous	LD • Group sessions at a community center with therapist present TD group • Therapist streamed via web during group sessions • Didactic education and interaction with group members • Participant’s presentation for long-term plan and feedback • Provided an imagery compact disc related to a weekly topic • Phone calls to encourage at-home practice	Web	• 3 months • Five 4 weekly group sessions- 25 min of didactic education during 4 sessions- 25 min of interaction with group members • Brief (<10 min) weekly phone calls	Wait list • Care as usual
Zachariae et al. (2018) [28]	RCT	Breast Cancer 133/122	CBTI (Tele-education) Asynchronous	• Automatically computed tailored recommendations • Online CBTI program and completing sleep diaries	Web	• 6 cores for 9 weeks • 1-week break for each core • 45–60 min for each core	Wait list • Care as usual
Kanera et al. (2017) [29]	RCT	Mixed cancer types 231/231	CBT (Tele- education) Asynchronous	• Personalized cancer aftercare intervention: generic information modules on the most common residual problems + feedback on their reported scores	Web	• 6 months • 8 modules	Wait list • Care as usual
Galiano-Castillo et al. (2017) [30]	RCT	Breast Cancer 39/37	Exercise program (Tele-education) Synchronous and asynchronous	• Tailored exercise program + individual supervision through a control platform • Instant messages, video conference sessions, telephone calls	Web	• 8 weeks • 3 sessions per week • 90 min per day • 24 exercise program sessions	Wait list • Care as usual (recommendations about PA using a written format)
Syrjala et al. (2018) [31]	RCT	Hematopoietic cell transplantation INSPIRE + PST 115/INSPIRE 114/Control 115	CBT + PST (Tele-education) Synchronous and asynchronous	INSPIRE + PST • INSPIRE - Psychological support, self-care tips and tools forum for survivor experiences, national and local resources • PST TH call - Problems and goal setting toward solutions INSPIRE • Only INSPIRE online intervention	Web	• 6 months • 7 INSPIRE sessions • 30 min, 3–7 PST process	Wait list • Care as usual
Lozano-Lozano et al. (2019) [33]	Quasi-experimental one group prepost	Breast Cancer 80	Lifestyle change (Tele-monitoring) Asynchronous	• Monitoring on PA (duration and intensity) and healthy eating (food and drink intake) + feedback • Self-recording with their own performance via the app	App	• 2 months • Daily recording	No
McCarroll et al. (2014) [34]	Quasi-experimental one group prepost	Breast Cancer/Endometrial Cancer 50	Lifestyle change (Tele-counseling) Asynchronous	• Exercise and nutrition counseling + real-time feedback component by the multidisciplinary team • Self-recording daily exercise and nutrition via the app	App	• 1 month • Daily recording	No
Lee et al. (2014) [32]	RCT	Breast Cancer 29/28	Lifestyle change (Tele-education) Asynchronous	• Assessment, education (tailored exercise and diet behavior) • Recommendation of action planning in dietary and exercise (goal setting, scheduling, keeping a diary), and automatic feedback (SMS module)	Web	• 3 months • Recording at least twice weekly • 5 education modules	• Usual care Educational booklet on exercise and diet

BCS: Breast Cancer Survivors, CBT: Cognitive Behavioral Therapy, CBTI: Cognitive Behavioral Therapy for Insomnia, INSPIRE: Internet-Based Survivorship Program with Information and Resources, LD: Live Delivery, MBT: Mind-Body Training, OT: Occupational Therapy, PA: Physical Activity, PST: Problem-Solving Therapy, RCT: Randomized Controlled Trial, RPE: Ratings of Perceived Exertion, h: Hour, STRIDE: Steps Toward Improving Diet and Exercise, TD: Telemedicine Delivery, TH: Telehealth, Tel: Telephone, Web: Website.

### 3.4. Characteristics of Telehealth Intervention 

The TH technology used in the studies is found in Table 2. The service delivery types in these studies were web-based (*n* = 10), telephone-based (*n* = 3), and via mobile applications (*n* = 2). The care models were tele-education (*n* = 11), tele-monitoring (*n* = 2), and tele-counseling (*n* = 2). TH types were synchronous (*n* = 6), asynchronous (*n* = 6), or both (*n* = 3).

### 3.5. Intervention Regime 

The frequency, duration, and length of interventions varied among the 15 studies. In terms of frequency, participation was daily (*n* = 4), three times a week (*n* = 2), twice a week (*n* = 1), weekly (*n* = 2), or monthly (*n* = 1). Five studies did not specify the frequency [23,25,27,28,30] The duration of each session ranged from 25 to 60 min. The length of interventions ranged from one to six months (Table 2). 

### 3.6. Outcome Measures 

The duration of the intervention from the baseline to the end was within 1–12 months, with 8 of 15 studies using ≥6 months as the final assessment (Table 3).

The outcome measures of the TH interventions included assessments of pain, fatigue, depression, anxiety, distress, mobility, anthropometry, physiological measures, muscle strength, physical activity level, exercise intensity, cognitive function, symptom burden, QOL, well-being, sleep quality, diet quality, self-efficacy or confidence, and motivational readiness (Table 3).

Other measures used included the rate of adherence to TH interventions, duration, satisfaction with delivery method or quality, usefulness, appropriateness of contents, barriers and facilitators to intervention contents, intervention effect according to participants’ age group and completion rate of the session (module) or assignment (Table 3). 

### 3.7. Intervention and Outcome

#### 3.7.1. Physical Activity (PA)

Three studies carried out PA interventions. These included tailored or individualized exercise programs, feedback through monitoring, and videoconference sessions [23,25,30]. Exercise improved QOL, physical fitness, muscle strength [23,25], mobility [30], and cognitive functions such as memory and attention [30]. It also reduced pain and fatigue [23,25] (Table 2 and Table 3).

#### 3.7.2. Symptom Self-Management 

Symptom self-management intervention programs consisted of the medical management of conditions or issues related to chronic conditions and programs for meaningful behavior change and maintenance [21,24]. Along with the education session, support materials (call or written format) were provided for the continuous support of changed behavior. After the intervention, there was significant relief of the symptom burden and improvement of QOL [21], reduction of pain, fatigue, and depression [24] (Table 2 and Table 3). 

#### 3.7.3. Lifestyle Behavior Change 

Four studies used lifestyle behavior change interventions based on exercise and diet behaviors using the web- or mobile-based applications. They consisted mainly of education or counseling, monitoring of physical activity (duration and intensity) and healthy eating (food and drink intake), and provided feedback [22,33,34,35]. Lifestyle change and support intervention had significant effects in improvements in moderate physical activity, self-efficacy for physical activity, QOL [22,33,35], and reduction in anthropometrics (body mass index, weight), positive changes in overall diet quality and consumption, and reduced severity of fatigue [35] (Table 2 and Table 3). 

#### 3.7.4. Psychosocial Intervention 

Six studies used psychosocial interventions. These included cognitive behavioral therapy for insomnia (CBTI) [20,28], CBT-alone [29], combined intervention of CBT and problem-solving therapy (PST) [26,31], and imagery-based behavioral therapy (IBT) [27] (Table 2 and Table 3). These psychosocial interventions commonly showed significant improvements in QOL, fatigue, and sleep quality. 

##### Cognitive Behavioral Therapy, Problem-Solving Therapy

Three studies used specific CBT modules, Kanker Nazorg Wijzer (KNW), or internet-based survivorship program with information and resources (INSPIRE) developed based on psychoeducation with a cognitive behavioral approach [26,29,31]. KNW consists mainly of educational modules and covers topics related to return to work, fatigue, anxiety and depression, social relationship and intimacy issues, physical activity, diet, and smoking cessation [35]. INSPIRE is a program for hematopoietic stem cell transplantation survivors with the aim of boosting health (cardiovascular, bone, and second cancer risks and recommendations), restoring energy (fatigue, muscle weakness, and inactivity), and renewing outlook (depression, distress, and social isolation) [36]. Problem-solving therapy (PST) is based on problem-solving, a component of psychological education, and it focuses on problem identification, solution finding, trying out solutions, and evaluating the result. The study that used CBT-alone [29] showed an increase in moderate physical activity, but did not show a significant intervention effect on vegetable consumption. A combination of CBT and PST showed a significant improvement in the emotional and social function of QOL, reduction of depression and fatigue [26], and distress [31] (Table 2 and Table 3).

##### Cognitive Behavioral Therapy for Insomnia (CBTI)

CBTI is an intervention aimed at improving sleep quality without the use of pills. Two studies used this intervention. It involved education on topics such as sleep restriction and stimulus control, cognitive restructuring, sleep hygiene and relapse prevention, and completion of the sleep diary by participants [20,28]. There was a significant improvement in sleep-related outcomes (e.g., sleep efficiency, sleep onset latency) and decreased insomnia severity. McCarthy et al. also reported a significant improvement in QOL, beliefs, and attitudes towards sleep and reduction of fatigue [20] (Table 2 and Table 3).

##### Mind–Body Training (MBT)

One study used mind–body training [27]. It focuses on the mind–body connection that helped in the identification of passive imagery (fear and loss of control), creation of active imagery (empowering, meaning–making themes), and practice of targeted imagery (imagining healthy physiological conditions). There was a significant improvement in fatigue, cognitive dysfunction, sleep disturbance, and QOL in the web-based intervention group compared with the waitlist. However, there were no differences between the web-based intervention group and in-person intervention group (Table 2 and Table 3). 

## 4. Discussion

This systematic review comprehensively evaluated the effects of TH interventions within the scope of OT on outcomes of occupational engagement. 

**Figure 2:**
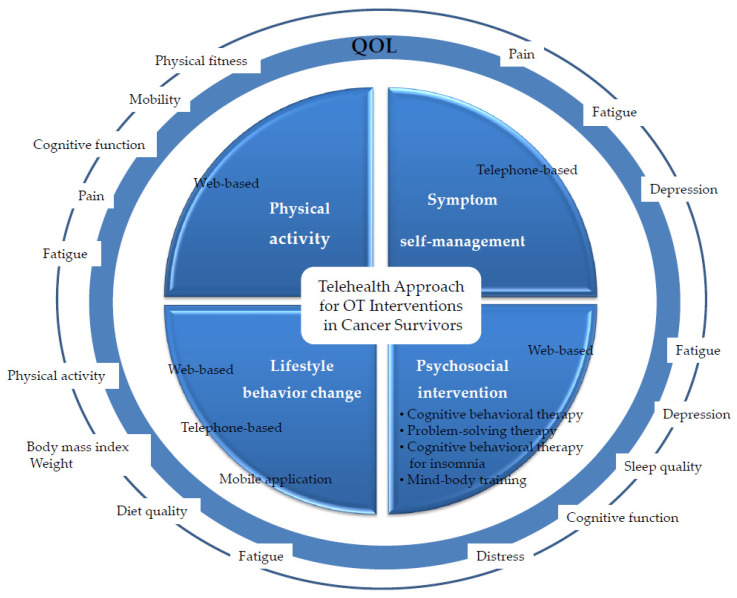
Summary of TH interventions in OT for cancer survivors.

This review provides evidence that PA interventions using TH technology for cancer survivors had a positive effect on physical function, cognitive function, cancer-related pain, fatigue, and QOL. The types of physical activity included resistance and/or aerobic exercise and walking (Figure 2). One study reported satisfaction with and adherence to PA interventions using the TH approach [23]. These interventions consisted of a battery of specific exercises, e.g., warm-up, resistance and aerobic exercise training, and cool-down. The effectiveness of PA interventions for cancer patients is known. They increase physical function, reduce cancer-related fatigue, and improve sleep quality and QOL regardless of the type or stage of cancer [37]. Spence et al. reported that PA is an effective approach for cancer patients or survivors regardless of the timing of cancer treatment [38]. OT practitioners can provide PA interventions using TH to incorporate PA into the survivors’ daily life for enhancing health, wellness, and QOL. 

Many cancer survivors experience symptoms such as fatigue, depression, hot flashes, breathing problems, pain, and sleep. There are specific symptoms that require management depending on the type of cancer [39]. In symptom self-management interventions included in the review, one study focused on the urinary tract, sexual organs, bowel, and general health for prostate cancer survivors. Another study addressed common concerns of breast cancer, health self-management techniques, and understanding of side effects. Both studies provided education and support via telephone. Symptom self-management interventions using a telephone had positive effects on relief of the symptom burden, improved the QOL, and reduced pain, fatigue, and depression [21,24]. OT practitioners can help clients engage in their occupational activities through symptom education and management by facilitating and supporting problem-solving skills using TH technology via a telephone.

Psychosocial interventions in the review were CBT, CBT-PST, CBTI, and MBT. Cancer survivors need to take an active role in managing their health and well-being. However, many survivors have low self-efficacy in managing fatigue and distress [40]. PST and CBT have been reported to improve survivors’ health and well-being management skills [41]. CBT is a problem-specific, goal-oriented approach that focuses on dealing with current problems such as inaccurate or negative thinking. PST focuses primarily on problem-solving skills including identifying problems, finding solutions, trying out solutions, and evaluating the results [31]. It has been reported that CBTI can lead to an improvement in sleep outcomes and a decrease in associated daytime symptoms in patients after cancer treatment [42]. MBT in the review was an image-based behavioral approach. It uses guided images to create a specific sensory experience for achieving clinical goals such as promoting the treatment of specific symptoms or overall well-being [43]. Hunter et al. reported that psychosocial components such as PST, CBT, and MBT are beneficial for survivors regardless of age or type or stage of cancer and can improve depression, anxiety, and QOL [37]. As a result of a combination of CBT and PST, there were significant improvements in depression and fatigue and distress reduction. In addition, Willems et al. showed a high adherence to TH interventions of 83.9% for 18 weeks [26]. In CBT-alone, moderate physical activity was improved in participants under the age of 57 [29]. CBTI improved sleep-related outcomes and QOL [20,28], and reduced fatigue [20]. Unlike face-to-face contacts, internet-based psychosocial interventions for cancer survivors overcome the limitations of accessibility. In this regard, the results of this review show the positive potential for psychosocial approaches using TH to improve survivors’ daily lives and occupational performance. 

Occupation-based theories can be complemented with health behavior change theories such as the health belief model, the transtheoretical or stages of change theory, and social cognitive theory. These will aid in evaluating the factors that lead to healthy behaviors and to develop behavior-based interventions that promote change [44]. Lifestyle behavior change interventions in the review addressed exercise and diet behavior using the web- or mobile-based applications. Two out of four studies were compared to control groups that provided usual care. The possibility of lifestyle behavior change interventions using TH as an effective delivery service was confirmed in this review. There were significant improvements in moderate physical activity, QOL [22,32,35], overall diet quality, and reduction of fatigue [35]. Kim et al. reported an adherence rate of >91% (exercise, diet) and helpfulness rate of >95% for TH interventions, showing the possibility of using TH as a lifestyle behavior change intervention [22]. 

There is a wide range of interventions that OT practitioners can provide for cancer survivors. The review confirmed that the PA TH interventions, symptom self-management, lifestyle behavior change, and psychosocial interventions improved the cognitive function, participation in PA, lifestyle change including dietary habits and QOL. They also reduced cancer-related pain, fatigue, depression, and anxiety of the survivors. Although the direct outcomes of occupational engagement excluding sleep could not be confirmed through this review, outcomes of factors affecting occupational engagement were confirmed. The limitations of this review arise from the study design and methods of the included studies such as small sample sizes, a control group setting that is difficult to compare with TH and in-person interventions. In the control group setting, most of the control groups were wait list groups, and interventions provided to them are related information and recommendations through written format. There were no comparative studies on the effects of in-person and TH intervention, adherence rate, and client’s satisfaction. In addition, in the case of chronic conditions, such as cancer survivors, it is important to verify the long-term effect of the intervention, but some studies in this review did not verify the sustained positive effect for a prolonged period of time after the completion of the intervention. The studies included in this review focused on the outcomes of cancer-related symptoms and QOL. No study reflected the perspective of OT such as occupational performance and social participation. However, this study identified the evidence that OT practitioners can apply TH within a unique professional perspective for cancer survivors and plan TH intervention programs addressing OT concerns. OT-specific research focusing on occupation-based interventions using TH for cancer survivors, interventions that cooperate with daily life, and outcomes of activities and participation will be needed in the future.

## 5. Conclusions

This review shows that a TH approach in the scope of OT for cancer survivors has positive therapeutic effects and offers the possibility of an alternative service delivery model of OT services to survivors. Although the direct outcomes of occupational engagement such as ADL, work, leisure, social participation, and sleep could not be confirmed through this review, outcomes of factors affecting occupational engagement were confirmed. Occupational therapy-specific research using TH interventions for cancer survivors will be needed in the future. These studies should focus on occupation-based interventions using TH, and outcomes of activities and participation. 

## Figures and Tables

**Table 3:** Summary of results of the included studies.

Author (Year)	Outcome Measures				Results	Other Effects
	Pre	Post		Assessment		
		T1	T2			
McCarthy et al. (2018) [20]	BL	6 weeks		• CSD (TST, SE, SOL, WASO, NA) • ISI • DBAS-16 • EORTC QLQ-C30 • R-PFS • HADS • MRS	• Significant improvements in sleep outcomes, including SE, SOL, WASO, TST, and NA (*p* < 0.001), QOL and daily functioning • Significant decreases in ISI and DBAS (*p* < 0.001) • Significant improvements in QOL (*p* < 0.001) and significant decrease in fatigue (*p* = 0.000) • No significant changes in anxiety (*p* = 0.417) or depression (*p* = 0.16) • Significant decrease in total menopausal symptoms (*p* < 0.001)	
Skolarus et al. (2019) [21]	BL	5 months	12 months	• EPIC •Confidence in self-management • Cancer control and outlook • PEPPI • Coping appraisal	• Significantly higher in all EPIC domain areas in the intervention group from baseline at 5 and 12 months (*p* < 0.001) but no significant differences between groups maintained at 12 months • Improvement in symptom focus area domains in the intervention group from baseline at 5 and 12 months • No differences in confidence in symptom self-management, cancer control and outlook, or PEPPI at 5 months • higher coping appraisal in the intervention group at 5 months (*p* = 0.02)	• Satisfaction rate with the program and intention to recommend: 80% or more
Kim et al. (2011) [22]	BL	3 months		•Stage of motivational readiness for exercise and diet • IPAQ • DQI • EORTC QLQ-C30 • HADS • BFI	• Significantly greater improvement in motivational readiness for exercise and diet, emotional functioning, fatigue, and depression in the intervention group • Significantly worsened DQI in the intervention group compared to that in the control group (*p* = 0.005)	• Adherence rate in IG: 94% for exercise, 91% for diet • Helpfulness rate in IG: 95% • Appropriateness of contents in IG: 96% for duration, 91% for frequency
Galiano-Castillo et al. (2016) [23]	BL	2 months	6 months	• EORTC QLQ-C30 • BPI • Isometric handgrip strength • Isometric abdominal strength • Isometric back strength • Lower body strength • R-PFS	• Significantly improved global health status, physical, role, cognitive functioning, and arm symptoms scores (all, *p* < 0.01) as well as pain severity (*p* = 0.001) and pain interference (*p* = 0.045) in the telerehabilitation group compared to the control group at 2 months • Significant improvements in affected and nonaffected side handgrip (both, *p* = 0.006); abdominal, back, and lower body strength (all, *p* < 0.01), and total fatigue (*p* < 0.001) in the telerehabilitation group at 2 months • These findings were maintained after 6-months of follow-up	• Adherence rate: 93.9% • Satisfaction rate in TH group: 97.8%
Meneses et al. (2018) [24]	BL	3 months	6 months	• SF-36 • CES-D	• Similar scores in physical and emotional well-being over time • Improved pain levels over 6 months (high effect size) • Improved fatigue scores at 3 months (moderate effect size) • Elevated depressive symptoms but no clinically significant change	
Frensham et al. (2018) [25]	BL	3 months	6 months	•Physical Activity using pedometer • Anthropometry (standing stretch stature, body weight, waist and hip girths) • Physiological measures (blood pressure) • 6MWT • SF-36	• Significant improvements in physical fitness (*p* < 0.01), systolic blood pressure (*p* < 0.01), diastolic blood pressure (*p* < 0.01), waist girth (*p* < 0.01), mental health (*p* < 0.05), social functioning (*p* < 0.01), and general health (*p* < 0.01) but an increase in bodily pain (*p* < 0.01) from baseline to 3 and 6 months	
Willems et al. (2017) [26]	BL	6 months		• EORTC QLQ-C30 • HADS • CIS	• Significant effect on increasing emotional (*p* = 0.022,) and social functioning (*p* = 0.011) and decreasing depression (*p* = 0.007) and fatigue (*p* = 0.020) in the intervention group but less strong evidence	• Average use of module: 2.22 ± 1.58 • Average time between first login and last use of a module: 10.67 ± 6.78 weeks • Adherence rate: 83.9%
Freeman et al. (2015) [27]	BL	1 months	3 months	• SF-36 • FACT-B • FACIT-F • FACT-Cog • FACIT-Sp-Ex • BSI-GSI • PSQI	• Significant improvement in fatigue, cognitive dysfunction, sleep disturbance, and health-related and breast cancer-related QOL in LD and TD compared to WL at 3 months (*p* < 0.01) • No differences between LD and TD at 3 months	
Zachariae et al. (2018) [28]	BL	9 weeks	15 weeks	• ISI • FACIT-F • Sleep diary (SOL, NA, WASO, EMA, TIB, TST, SE, sleep aids)	• Statistically significant improvement in all sleep-related outcomes from pre- to postintervention (*p* < 0.02) • Effect sizes (Cohen’s d) ranged from 0.33 (95% CI = 0.06 to 0.61) for wake after sleep onset to 1.17 (95% CI = 0.87 to 1.47) for insomnia severity • Maintained improvements for outcomes measured at follow-up (d = 0.66–1.10)	• Number of cores completed in TH group: 4.1 ± 2.5/6 • No differences between groups in the mean number of completed sleep diaries at baseline or postintervention
Kanera et al. (2017) [29]	BL	6 months	12 months	• SQUASH • Vegetable consumption (number of days per week, number of vegetable servings per day)	• Significant effect after 12 months for moderate physical activity (complete cases: *p* = 0.010; intention-to-treat: *p* = 0.011) in the intervention group • No significant intervention effect after 12 months for vegetable consumption (complete cases: *p* = 0.121; intention-to-treat: *p* = 0.132) in the intervention group	• Intervention effect among participants aged younger than 57 years (*p* = 0.000)
Galiano-Castillo et al. (2017) [30]	BL	8 weeks	6 months	• 6MWT • ACT • TMT	• Significantly improved distances (*p* < 0.001) and percentages of predicted 6 min walk test (*p* < 0.001) in the intervention group compared to the control; findings maintained after 6 months (*p* = 0.001; *p* = 0.002) • Significant improvement in the number of consonants recalled in the intervention group compared to that in the control group (*p* = 0.04); finding maintained after 6 months (*p* = 0.02)	
Syrjala et al. (2018) [31]	BL	6 months		• CTXD • SCL-90-R • SF-36 • FSI	• No differences in the mean change in aggregated outcomes in distress, depression, fatigue, and physical function among three groups (*p* = 0.30) • Analyses of participants with impaired scores showed significantly improved distress for INSPIRE + PST compared to controls (*p* = 0.032) • A trend toward improvement in distress in the INSPIRE alone group (*p* = 0.075), no differences between intervention arms and controls in rates of change in depressive symptoms, fatigue, or physical functioning (RR 0.6 to 1.4) • Marginal benefit in distress with the addition of TH PST, particularly for those who viewed the website or were age 40 years or older	
Lozano- Lozano et al. (2019) [33]	BL	2 months		• EORTC QLQ-C30 • SEPA • PA using accelerometry • Anthropometrics (BMI, percentage of fat mass, bone mineral density, height, weight)	• Significant improvements in QOL (*p* < 0.001): moderate to large effects • Significant improvements in SEPA scores (*p* < 0.001) • Daily moderate-to-vigorous PA (*p* = 0.04) • Reduced body weight and BMI (both, *p* < 0.001).	• Use rate: 76%, • Adoption rate: 69% • Patients’ perception of app quality: satisfaction (positive NPS) • Barriers: absence of some food items • Facilitators: relevant information to the patient
McCarroll et al. (2014) [34]	BL	1 months		• FACT-G • WEL •Anthropometrics (BMI, weight, waist circumference) • Daily food intake • PA minutes	• Significant reductions in anthropometric factors including weight, BMI, and waist circumference (*p* < 0.0006) between pre- and postintervention • Significant improvement in total WEL score (*p* = 0.043) between pre- and postintervention • No significant differences in FACT-G, macronutrient consumption, and PA patterns	
Lee et al. (2014) [32]	BL	3 months		• Intensity aerobic exercise • Intake of F&V • DQI • EORTC QLQ-C30 • HADS • BFI • Stage of change • Perceived self-efficacy	• Significantly increased moderate-intensity aerobic exercise for at least 150 min per week (*p* < 0.0001) and eating five servings of F&V per day (*p* = 0.001) in the intervention group • Greater improvement in overall diet quality in the intervention group compared to that in the control group (*p* = 0.001) • Significantly higher proportions of patients in whom protein and calcium intake met the RDA in the intervention group than those in the control group (respectively, *p* = 0.016, 0.003) • Significantly improved physical functioning (*p* = 0.023) and appetite loss (*p* = 0.034) of QOL, severity of fatigue (*p* = 0.032) in the intervention group compared to those in the control group • Significant improvement in stage of behavior change for exercise (*p* < 0.0001) and F&V consumption (*p* = 0.029) in the intervention group than those in the control group • Significant difference in self-efficacy for exercise management and F&V intake (*p* = 0.024 and *p* = 0.023, respectively)	• Adherence rate: 89% • Positive evaluations of the contents, the IT-supported delivery method, and the system’s usefulness

ACT: Auditory Consonant Trigrams, BFI: Brief Fatigue Inventory, BL: Baseline, BMI: body mass index, BPI: Brief Pain Inventory, BSI-GSI: Brief Symptom Inventory-Global Severity Index, CES-D: Center for Epidemiologic Studies Depression Scale, CI: confidence interval, CIS: Checklist Individual Strength, CSD: Consensus Sleep Diary, CTXD: Cancer and Treatment Distress, DBAS-16: Dysfunctional Beliefs and Attitudes about Sleep-16, DQI: diet quality index, EMA: early morning awakening, EORTC QLQ-C30: European Organization for Research and Treatment of Cancer Quality of Life Questionnaire Core 30, EPIC: Expanded Prostate Cancer Index Composite-26, FACIT-F: Functional Assessment of Cancer Therapy-Fatigue Scale, FACIT-Sp-Ex: Functional Assessment of Chronic Illness Therapy Spiritual Well-Being Expanded Scale, FACT-B: Functional Assessment of Cancer Therapy-Breast, FACT-Cog: Functional Assessment of Cancer Therapy-Cognition Scale, FACT-G: Functional Assessment of Cancer Therapy-General, FSI: Fatigue Symptom Inventory, F&V: fruits and vegetables, HADS: Hospital Anxiety and Depression Scale, IG: Intervention Group, IPAQ: International Physical Activity Questionnaire, ISI: Insomnia Severity Index, IT: Information Technology, LD: Live Delivery, MRS: Menopause Rating Scale, NA: number of nocturnal awakenings, NPS: Net Promoter Score, PA: physical activity, PEPPI: Perceived Efficacy in Patient-Physician Interactions, PSQI: Pittsburgh Sleep Quality Index, QOL: Quality of Life, RDA: Recommended Daily Allowances, RPE: Ratings of Perceived Exertion scale, R-PFS: Revised-Piper Fatigue Scale-revised, RR: Relative Risks, SCL-90-R: Symptom Checklist-90-R depression scale, SE: sleep efficiency, SF-36: Short-Form 36 Health Survey, SEPA: Self-Efficacy scale for Physical Activity, SOL: Sleep Onset Latency, SQUASH: Short Questionnaire to Assess Health Enhancing Physical Activity, TD: Telemedicine Delivery, TIB: Time In Bed, TMT: Trail Making Test, TST: Total Sleep Time, WASO: Wake After Sleep Onset, WEL: Weight Efficacy Lifestyle Questionnaire, WL: Waitlist, 6MWT: 6-Min Walk Test.
